# Anorectal tuberculosis coexisting with adenocarcinoma: an unusual association

**DOI:** 10.1186/1757-1626-2-143

**Published:** 2009-09-29

**Authors:** Sudeep Khaniya, Rabin Koirala, Vikal Chandra Shakya, Shailesh Adhikary, Rajendra Regmi, Sagar Raj Pandey, Chandra Shekhar Agrawal

**Affiliations:** 1 Department of Surgery, B. P. Koirala Institute of Health Sciences, Dharan, Nepal; 2 Department of Pathology, B. P. Koirala Institute of Health Sciences, Dharan, Nepal

## Abstract

**Introduction:**

Tuberculosis affecting the anorectum is an uncommon extra-pulmonary form of the disease, and its association with malignancy is highly unusual.

**Case report:**

A 35 year lady presented with lower gastrointestinal bleed, altered bowel habit and significant weight loss. On examination, she had nodular stricture in the lower rectum, with friable mucosa, bleeding easily on touch. With the diagnosis of carcinoma lower rectum, she underwent abdomino-perineal resection of the growth. The histopathological examination revealed carcinoma rectum with coexisting tuberculosis.

**Conclusion:**

The aetiological association between the tuberculosis and anorectal cancer is a matter of debate. However, the treating surgeon should be aware of this association, to avoid confusion and delay in the management.

## Introduction

Intestinal tuberculosis is primarily located in the ileoceacal region, with anorectal tuberculosis being an uncommon site even in the endemic region [1]. The occurrence of tuberculosis with malignant anorectal lesion is further rare, though this association has been described in the literature with questionable aetiological relationship [2,3].

## Case report

**Figure 1:**
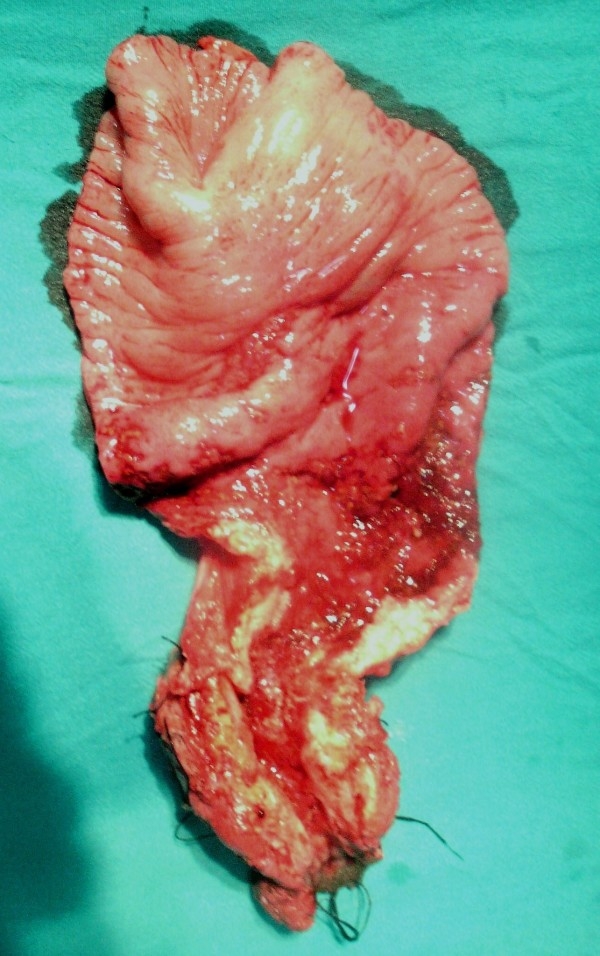
**Specimen of abdominoperineal resection showing hypertrophic ulcerated stricture involving the lower rectum and upper anal region**.

**Figure 2:**
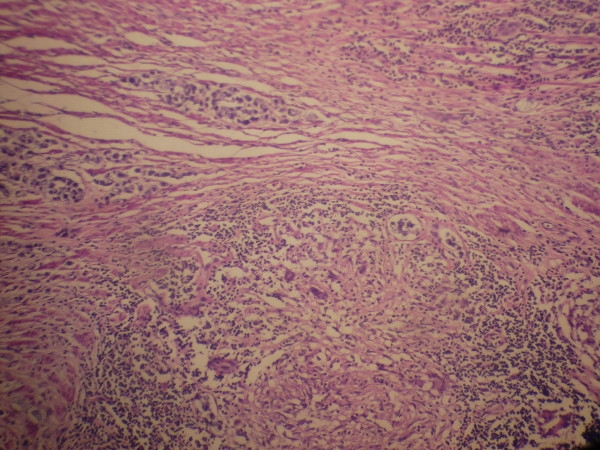
**Histopathological examination showing signet-ring adenocarcinoma involving the muscular layer of the rectum along with tubercular granuloma**.

**Figure 3:**
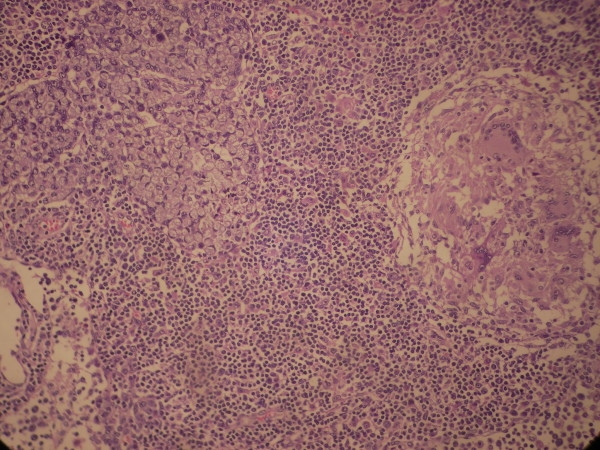
**Histopathological examination of the lymph node showing metastatic deposits of signet ring adenocarcinoma and tubercular granuloma**.

A 35 year Mongolian lady presented with history of abdominal pain, bleeding per rectum, gradually worsening constipation along with weight loss of 7 kg over a period of 9 months. There was no history of pulmonary symptoms such as cough, haemoptysis or shortness of breath. Past history of tuberculosis or contacts with diagnosed case of tuberculosis was absent. On examination multiple faeculoma were palpable in the abdomen, rectal examination showed tight, nodular stricture in the lower rectum. The sigmidoscope could not be passed beyond the stricture, and the mucosa was friable and bleeding easily on touch. Rectal biopsy revealed dysplastic cells with features of non specific chronic inflammation on 2 occasions. No abnormality was detected in chest X-ray. Contrast enhanced CT scan of the abdomen and pelvis showed thickened rectal wall alongwith stricture at the lower rectum. Laparotomy revealed dilated and edematous upper rectum and sigmoid colon with multiple nodules on the surface, with presence of hard stricture in the lower rectum. Patient underwent abdomino-perineal resection, resected specimen showing hypertrophic ulcerated stricture involving the lower rectum and upper anal region with enlarged multiple perirectal lymph nodes. (Fig. 1) Histopathological examination showed signet-ring adenocarcinoma infiltrating to the serosa with caseating granulomas containing epitheloid cells and Langhan's giant cells. (Fig. 2) The resected lymph nodes showed both metastatic deposit and tuberculous granulomas.(Fig. 3) The patient received 6 months of anti-tuberculous therapy followed by chemotherapy (5-flurouracil and leucovorin), and is symptom-free at the end of one year.

## Discussion

Colonic tuberculosis is a rare extrapulmonary form of the disease, comprising only 3-4% of intestinal tuberculosis [4]. Even in colon, rectum is unusual site for the tuberculosis, and its association with malignancy is more uncommon. The association of these two conditions has been a matter of debate. The coexistence of tuberculosis and carcinoma in the colon may be simply a coincidence [2]. On the other hand, one disease process might have initiated the second. Some authors have postulated that cancer of the colon is the primary lesion, followed by secondary infection of the tuberculous bacilli in the malignant ulcer of the rectum, which might have been facilitated by luminal obstruction, impaired cellular immunity and loss of mucosal barrier [3,5]. However, Mishra et al have suggested that the long standing tuberculous ulcer may be carcinogenic, with development of invasive carcinoma similar to Crohn's disease and Schistosomiasis [3]. The presentation of the diseases, clinically or during laparotomy, mimic each other and may lead to misdiagnosis of either lesion. Therefore, while managing similar cases, even in the West, considering the rise of HIV infection and immigration of people from the endemic regions, awareness of this association should be borne in mind [6].

## Abbreviations

CT: Computed Tomography

## Consent

Informed written consent for the publication of the article and accompanying images was obtained from the patient. One copy of the consent form is available for review by the Editor.

## Competing interests

The authors declare that they have no competing interests.

## Authors' contributions

SK and RK made substantial contributions to concept and design of the article. VCS, RR and SRP were involved in the acquisition of materials. CSA and SA contributed significantly in the critical revision and drafting of the manuscript. All authors read and approved the final version of the manuscript.
